# Dejar morir: la trágica gestión de la pandemia de la covid-19 en Brasil

**DOI:** 10.1590/S0104-59702023000100050

**Published:** 2023-09-18

**Authors:** Claudia Agostoni

**Affiliations:** i Instituto de Investigaciones Históricas Universidad Nacional Autónoma de México agostoni@unam.mx Instituto de Investigaciones Históricas/Universidad Nacional Autónoma de México. agostoni@unam.mx

**Figure:**
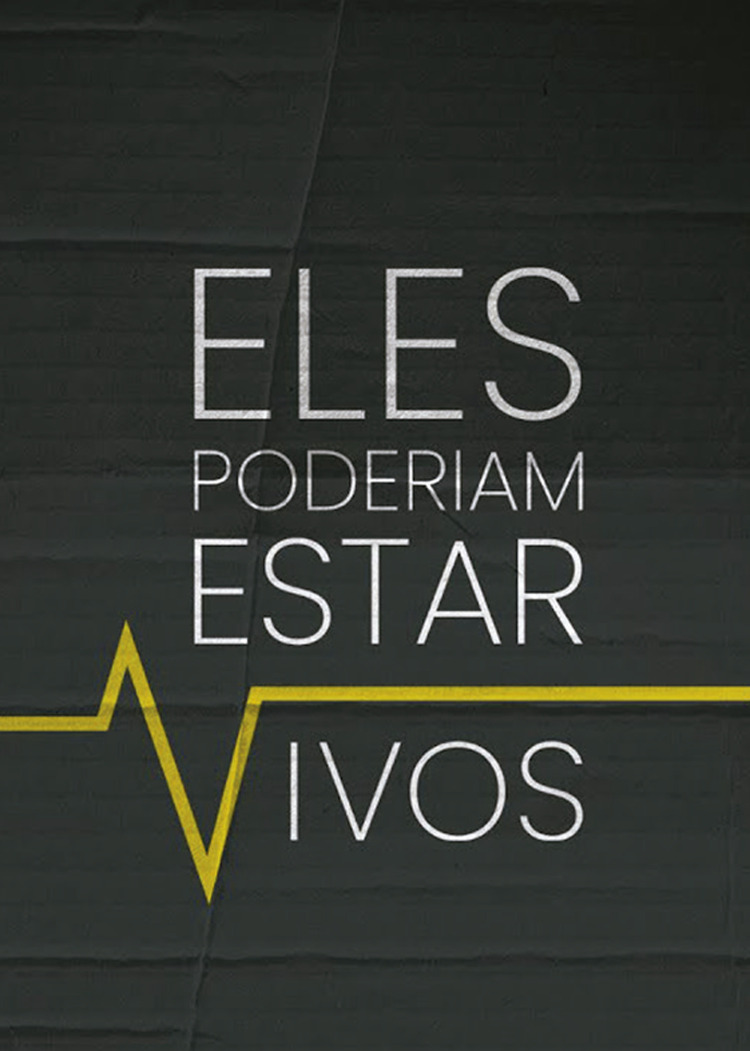
ELES PODERIAM ESTAR VIVOS. Dirección: Gabriel y Lucas Mesquita. Brasil: documental independiente, 2022. 128 min.

El documental independiente *Eles poderiam estar vivos* (2022), escrito, dirigido y producido por los hermanos Lucas y Gabriel Mesquita, es un detallado y contundente testimonio de la trágica gestión de la pandemia de la covid-19 en Brasil. Uno de sus argumentos centrales es que la estrategia privilegiada por el gobierno autoritario de Jair Bolsonaro fue la deliberada propagación del virus y que ello provocó la muerte de por los menos la mitad de las 680 mil personas que se estima que fallecieron entre 2020 y 2022.

El documental, un notable registro y testimonio de nuestro tiempo, inicia con una importante afirmación de Deisy de Freitas de Lima Ventura, profesora titular de ética de la Facultad de Salud Pública de la Universidade de São Paulo: la pandemia de la covid-19 no fue una sorpresa para la comunidad médica y científica brasileña y, tampoco para la comunidad médica internacional, ya que desde tiempo atrás se contaba con sólidos estudios y con contundentes informes científicos relativos a una próxima propagación pandémica de algún coronavirus. Ventura acota que lo que no se sabía era en qué momento iniciaría, o de qué virus se trataría, y destaca que Brasil contaba con la experiencia histórica, con el personal sanitario y con un sólido Sistema Único de Saúde (SUS), al igual que con comprobadas estrategias de salud pública para afrontar diferentes emergencias sanitarias. Por ello, es importante remarcar que Brasil sí tenía los recursos, las instituciones, el personal sanitario y, una larga y sólida trayectoria histórica en salud pública. Al respecto se pueden mencionar sus consolidados servicios de atención primaria y casi cincuenta años de experiencia organizando exitosos programas y campañas de vacunación, siendo que Zé Gotinha, un personaje clave de las campañas y programas de vacunación, forma desde tiempo atrás parte del patrimonio nacional. Esos y otros elementos, propulsores de notables transformaciones y mejoras en el ámbito de la salud pública durante los siglos XX y XXI, aunque han sido cuidadosa y detalladamente estudiados en diferentes investigaciones históricas, fueron cotidianamente saboteados, debilitados y con ello olvidados por el gobierno de Jair Bolsonaro y por los actores e instituciones afines a su administración.

El documental también presenta, desde una perspectiva multidisciplinaria, los testimonios de destacados especialistas en salud pública, epidemiología, psiquiatría, leyes y ética de la salud pública. Incluye múltiples fotografías, ilustraciones y caricaturas publicadas en la prensa brasileña realizadas por Carlos Latuff, Paula Villar y Cris Vector, así como íntimos y desgarradores relatos de los familiares de las víctimas de la errática gestión de la pandemia. Esas entrevistas, narraciones, imágenes y secuencias, son cohesionadas o articuladas a partir de la inserción de una detallada línea del tiempo, la que muestra que si bien la gestión de la pandemia inicio por buen camino el 6 de febrero de 2020, la estrategia rápidamente cambió de curso. Se afirma que fue a partir del 23 de marzo, al promulgarse la Resolução de Diretoria Colegiada n. 354, la que eliminó la obligatoriedad de presentar una receta médica para la compra y uso de la cloroquina y de la hidroxicloroquina, cuando se favoreció e impulsó el uso de dos medicamentos carentes de ensayos clínicos controlados que demostraran que sí eran eficaces para el tratamiento de los pacientes con la covid-19. Lo anterior no solo representó el desdén del gobierno brasileño hacia las directrices científicas internacionales y hacia la Organización Mundial de la Salud. También es un factor que constata que lo que se buscó fue la deliberada estrategia de inmunidad de rebaño por contagio, lo que se sustentó en ideas falsas y éticamente cuestionables en aras de preservar el funcionamiento de la economía. El documental también detalla cómo otras leyes y decretos favorecieron el contagio deliberado de la población. Al respecto se pueden mencionar los del 25 de marzo y 11 de mayo de 2020 que determinaron que todas las actividades religiosas y que las loterías se considerarían como actividades económicas esenciales, lo mismo que los salones de belleza, las peluquerías, las escuelas de deportes en todas sus modalidades y, las actividades industriales.

La deliberada estrategia del contagio masivo para adquirir una “inmunidad de rebaño” es sobre lo que reflexionan la ya mencionada Deisy Ventura, al igual que Fernando Aith y Luana Araújo, infectológa que formó parte y quien testificó en la Comisión de Investigación Parlamentaria (CPI) de Brasil sobre la covid-19 (CPI de la covid-19, 27 abr. 2021 a 26 oct. 2021) y Arthur Chioro, ex ministro de Salud, entre otras de las personas entrevistadas. Ellos explican cómo lo anterior se acompañó de una sistemática minimización de los peligros, de una intensa campaña de desinformación y de noticias falsas que provocaron que la mayor parte de los fallecimientos se registraran entre los sectores más pobres y vulnerables del país. Esa estrategia, tal y como se constata a lo largo del documental, representó una confrontación con la Organización Mundial de la Salud (OMS), con los sustentos históricos y éticos de la salud pública y, con las instituciones y actores nacionales e internacionales de la salud pública. Considero particularmente importante mencionar que, de acuerdo con la OMS (12 oct. 2020, s.p.), “nunca en la historia de la salud pública se ha recurrido a la inmunidad colectiva como una estrategia para responder a un brote, y mucho menos a una pandemia”. Ello plantearía problemas científicos y éticos.

[D]ejar que el virus circule descontroladamente supone infecciones, sufrimientos y muertes innecesarios … Permitir que un virus peligroso, cuyos mecanismos no conocemos cabalmente, circule sin control es algo contrario a la ética. Ésa no es una opción … No es una elección entre dejar que el virus circule libremente o paralizar nuestras sociedades (Ventura y Costa Bueno, 2021, p. 442).

Sin embargo, lo que se privilegió en Brasil fue una peligrosa deformación de la realidad.

La deliberada y descontrolada propagación del virus se acompañó de una sistemática y tenaz obstrucción a las respuestas que los estados federales y los municipios estaban procurando implementar para contener los contagios, de una incesante descalificación de los beneficios del distanciamiento social y del uso de las mascarillas, de una intensa campaña de desinformación relativa a las vacunas y de la asociación que Bolsonaro estableció entre contagio, cobardía y debilidad. Además, es frente a la reiterada minimización de la pandemia, la que fue constantemente calificada por Bolsonaro como una *gripezinha* o un *resfriadinho*, que el documental presenta las historias de vida de hombres y mujeres que perdieron a sus familiares y amistades a causa de las medidas y estrategias gubernamentales, relatos que transmiten una profunda sensación de abandono, tristeza, enojo e incredulidad.

Las desgarradoras narraciones de los familiares y amigos de todas las personas que no debían morir, es un impresionante retrato de la tragedia humana que se alimentó de la ceguera de los gobernantes y por causa de la falsa dicotomía entre economía y salud. El documental es un testimonio fundamental de nuestro tiempo para comprender cómo se enfrentó la pandemia en el Brasil de Jair Bolsonaro y será un recurso de primera importancia para todos los interesados en el análisis histórico de la salud pública en general, y de la pandemia de la covid-19, en particular. Concluyo insertando las siguientes estrofas de la producción musical *Eles poderiam estar vivos* en voz de Crônica Mendes (Eles poderiam…, 26 ago. 2022), con lo que finaliza el documental:^1^

Ellos podrían estar vivos

Ellos deberían estar vivos

Por culpa de ellos, no están vivos

¿Cuántos sueños quedaron destruidos?

Ellos podrían estar vivos

Ellos deberían estar vivos

Por culpa de ellos, no están vivos

¿Cuántos sueños quedaron destruidos?

Familias fueron dilaceradas

Tratando de salvar la economía

Se diseminó el virus, enseguida se acabó todo

Todo el mundo morirá un día

Pero los muertos no consumen

No pagan impuestos

Se destruyó la economía

E incluso se mató al pueblo

## NOTA

1 En traducción libre.
